# *Anaplasma phagocytophilum* invasin AipB interacts with the β2 integrin β-subunit CD18 to orchestrate infection

**DOI:** 10.1128/mbio.03853-25

**Published:** 2026-02-24

**Authors:** Mary Clark H. Lind, Waheeda A. Naimi, Jason R. Hunt, Jerilyn R. Izac, Richard T. Marconi, Jason A. Carlyon

**Affiliations:** 1 Department of Microbiology and Immunology, School of Medicine, Virginia Commonwealth University Medical Center 72054, Richmond, Virginia, USA; Massachusetts Institute of Technology, Cambridge, Massachusetts, USA

**Keywords:** *Anaplasma*, invasin, adhesin, CD18, β2 integrin, obligate intracellular bacteria, *Anaplasmataceae*, *Rickettsiales*, host-pathogen interactions

## Abstract

*Anaplasma phagocytophilum* is an obligate intracellular bacterium that invades neutrophils to cause the potentially severe infection, human granulocytic anaplasmosis. The *A. phagocytophilum* invasin-host cell receptor interactions that facilitate bacterial uptake and hence disease are incompletely defined. Here, we show that EPHNCH_1611, which we refer to as *A. phagocytophilum* invasion protein B (AipB), mediates bacterial entry but not adhesion. The AipB host cell-interacting domain was narrowed down to amino acids 109 to 129 and is conserved among *Anaplasma* and *Ehrlichia* species. AipB_109-123_ antisera reduced *A. phagocytophilum* infection of host cells by 28%. Combining anti-AipB_109-123_ with antisera specific for the receptor binding domains of previously identified invasins AipA, Asp14, and OmpA reduced infection by 93%, suggesting a synergistic relationship among these surface proteins. Immunizing mice against AipB_109-123_ alone yielded partial protection from *A. phagocytophilum* challenge that rivaled the protection achieved by immunizing against it together with other invasin binding domains. A yeast two-hybrid screen identified the N-terminal domain of CD18, the β2 integrin β-subunit found on the neutrophil surface, as an AipB binding partner. The interaction was validated by co-immunoprecipitation. siRNA knockdown of CD18 reduced permissiveness to *A. phagocytophilum* infection. Antibodies targeting the CD18 N-terminus inhibited *A. phagocytophilum* infection of, but not adherence to, HL-60 cells and human neutrophils. These results demonstrate that the AipB-CD18 interaction is critical for *A. phagocytophilum* infection *in vitro* and *in vivo*, further dissecting the multifaceted mechanism by which *A. phagocytophilum* invades host cells.

**IMPORTANCE:**

*Anaplasma phagocytophilum* causes human granulocytic anaplasmosis, an emerging tick-borne infection for which there is no vaccine and limited treatments. Although *A. phagocytophilum* absolutely relies on invading host neutrophils to survive and cause disease, the microbe-host cell interactions that predicate infection are inadequately defined. We found that the bacterium uses its outer surface protein AipB to engage CD18 as a critical invasion step. An AipB peptide that is conserved among its homologs in other *Anaplasmataceae* pathogens is responsible for binding CD18 and can be targeted by antibody to inhibit infection *in vitro* and *in vivo*. However, blocking of *A. phagocytophilum* infection is most effectively achieved by targeting this AipB peptide together with the functional domains of other invasins. AipB is the first rickettsial invasin identified to interact with a β2 integrin, is key for pathogenesis, and could be targeted to protect against diseases caused by *A. phagocytophilum* and other *Anaplasmataceae* bacteria.

## INTRODUCTION

Obligate intracellular bacteria threaten public health worldwide. These microorganisms’ survival and hence pathogenesis are predicated on the ability to invade eukaryotic cells. Many do so by utilizing outer membrane proteins called invasins that engage specific host cell surface receptors. Dissecting invasin-receptor interactions improves fundamental understanding of obligate intracellular bacterial pathobiology and can thereby benefit development of prophylactics or vaccines against the diseases that they cause. *Anaplasma phagocytophilum* is an obligate intracellular bacterium and etiologic agent of human granulocytic anaplasmosis (HGA), the second most common tick-transmitted disease in the United States. HGA occurs on every continent except Antarctica, although cases in the United States tend to be more frequent and serious in nature (1–3). HGA is an acute febrile illness that can be accompanied by chills, headache, myalgia, and malaise. Frequent laboratory abnormalities include leukopenia, thrombocytopenia, anemia, and transaminitis. Complications can include shock, sepsis, disseminated intravascular coagulation, inflammatory syndromes, renal failure, hemorrhages, and rhabdomyolysis (4, 5). Hospitalization is required for 36% of cases, with a 14.6 day average stay and intensive care unit admittance for 7% (4, 6–8). A review of cases from 2002 to 2021 found the mortality rate to be 4.2% in immunocompetent and 18.2% in immunocompromised individuals (7). *A. phagocytophilum* is susceptible to tetracyclines, but there is no vaccine to prevent infection (9, 10). The risk of fatal or opportunistic infections due to HGA is greater when antibiotic therapy is delayed (7). Reliable diagnostic assays are lacking, which, when combined with its non-specific onset and risk for becoming severe if not treated early, make HGA a serious concern (4).

*A. phagocytophilum* is transmitted by *Ixodes* ticks and infects peripherally circulating neutrophils (5). In tick and myeloid cells, the bacterium progresses through a biphasic developmental cycle in which an infectious dense-cored (DC) form induces its receptor-mediated uptake into an endosomal compartment, where it converts to the non-infectious reticulate cell (RC) morphotype that divides by binary fission to fill the *A. phagocytophilum* vacuole (ApV) with bacteria. RCs transition into DCs, and ApVs undergo exocytosis to release infectious DC progeny (11–14). As the DC form is non-replicative and sensitive to the extracellular environment, the ability to bind and invade host cells is crucial. Three bacterial surface proteins—outer membrane protein A (OmpA), 14-kDa *A. phagocytophilum* surface protein (Asp14), *A. phagocytophilum* invasion protein A (AipA)—and their cognate receptors are key for invasion of neutrophils and promyelocytic HL-60 cells (15–20). OmpA residues 59–74 (OmpA_59-74_) bind α2,3-sialic acid and α1,3-fucose of the sialyl Lewis x (sLe^x^) glycan that caps P-selectin glycoprotein ligand-1 (PSGL-1) to dock *A. phagocytophilum* to the host cell surface (19). Asp14 residues 113–124 (Asp14_113-124_) interact with protein disulfide isomerase (PDI) to promote thiol reduction of bacterial surface proteins as a critical step in the entry process (15, 19). AipA amino acids 9–21 (AipA_9-21_) engage CD13 to initiate Src kinase signaling that also facilitates *Anaplasma* uptake (17, 20). PDI and CD13 are enriched in lipid rafts (15, 17, 21–24), which is consistent with the importance of lipid rafts for *A. phagocytophilum* invasion (25). An antisera cocktail that blocks the OmpA, Asp14, and AipA binding domains together inhibits infection considerably better than targeting them individually or pairwise. Yet, this cocktail inhibits infection by only 70% (19), hinting at the likely involvement of additional unidentified invasins.

EPHNCH_1611 (GenBank: KJV59337.1) is a 17.9-kDa (163-amino acid) *A. phagocytophilum* str. NCH-1 surface protein that is unique to *Anaplasma* and *Ehrlichia* species (26). When first studied, it was referred to as APH_1235 per its annotation in the *A. phagocytophilum* str. HZ genome (27). It is robustly induced during RC-to-DC transition and the tick transmission blood meal (26, 28). Cultivation of *A. phagocytophilum*-infected HL-60 cells in the presence of antiserum against this protein over a 4 day period significantly reduces the bacterial load (28). While these data suggest that EPHNCH_1611 potentially contributes to *A. phagocytophilum* infection, its functional role and targeted receptor are unknown.

β2 integrins are heterodimeric transmembrane receptors found exclusively on leukocytes (29, 30). They are composed of an α-subunit (CD11a–d) non-covalently bound to a β-subunit (CD18) (29). The α-subunit determines the function, ligand affinity, and cell-specific expression of β2 integrins, while the β-subunit conveys intracellular signaling (29–32). CD11b/CD18 (Mac-1) is constitutively expressed on the neutrophil surface and plays roles in phagocytosis, neutrophil aggregation, degranulation, adherence to the endothelium, and generation of reactive oxygen species (ROS) (24, 33, 34). Of the β2 integrins, Mac-1 is the most promiscuous, binding over 40 ligands (35). β2 integrins transition between low (bent conformation) and high affinity (extended-open conformation) states, which can be induced by an extracellular ligand triggering outside-in activation or by an intracellular signal inducing inside-out activation (35). Although β2 integrins are crucial for host defense, a few pathogens are known to exploit them for entry (36–38). *A. phagocytophilum* binding to human neutrophils under shear stress upregulates surface expression of high-affinity CD18 (39, 40). Similarly, infected neutrophils recovered from laboratory mice exhibit elevated Mac-1 surface levels (34). *A. phagocytophilum* cellular invasion elicits signaling associated with β2 integrin engagement (17, 24, 25, 39, 41–44). Finally, binding of the bacterium to neutrophils inhibits their ability to arrest on endothelium (39, 40), which could be due to it docking to and thereby occluding CD18 from interacting with endothelial cells. Whether β2 integrin is an *A. phagocytophilum* receptor for entry has yet to be directly shown.

In this study, we demonstrate that EPHNCH_1611, hereafter referred to as AipB (*A. phagocytophilum* invasion protein B), engages CD18 to promote *Anaplasma* entry. We found that the AipB domain that is essential for infection lies within amino acid residues 109 to 129 and is highly conserved among *Anaplasmataceae* pathogens. Likewise, we identified the N-terminal portion of CD18 that is required for *A. phagocytophilum* infection of host cells. We defined the relevance of AipB alone and in concert with Asp14 and AipA to *A. phagocytophilum* infection *in vivo*. We confirmed that targeting AipB_109-123_ together with OmpA_59-74_, Asp14_113-124_, and AipA_9-21_ improves the antisera cocktail’s blocking ability to nearly abolish the bacterium’s cellular invasion. Overall, we report the first obligate intracellular bacterium that interacts with CD18 to facilitate infection of neutrophils and fill a key knowledge gap in holistically understanding the myriad of host-pathogen interactions that mediate *A. phagocytophilum* infection.

## RESULTS

### AipB is an *A. phagocytophilum* invasion

**Fig 1:**
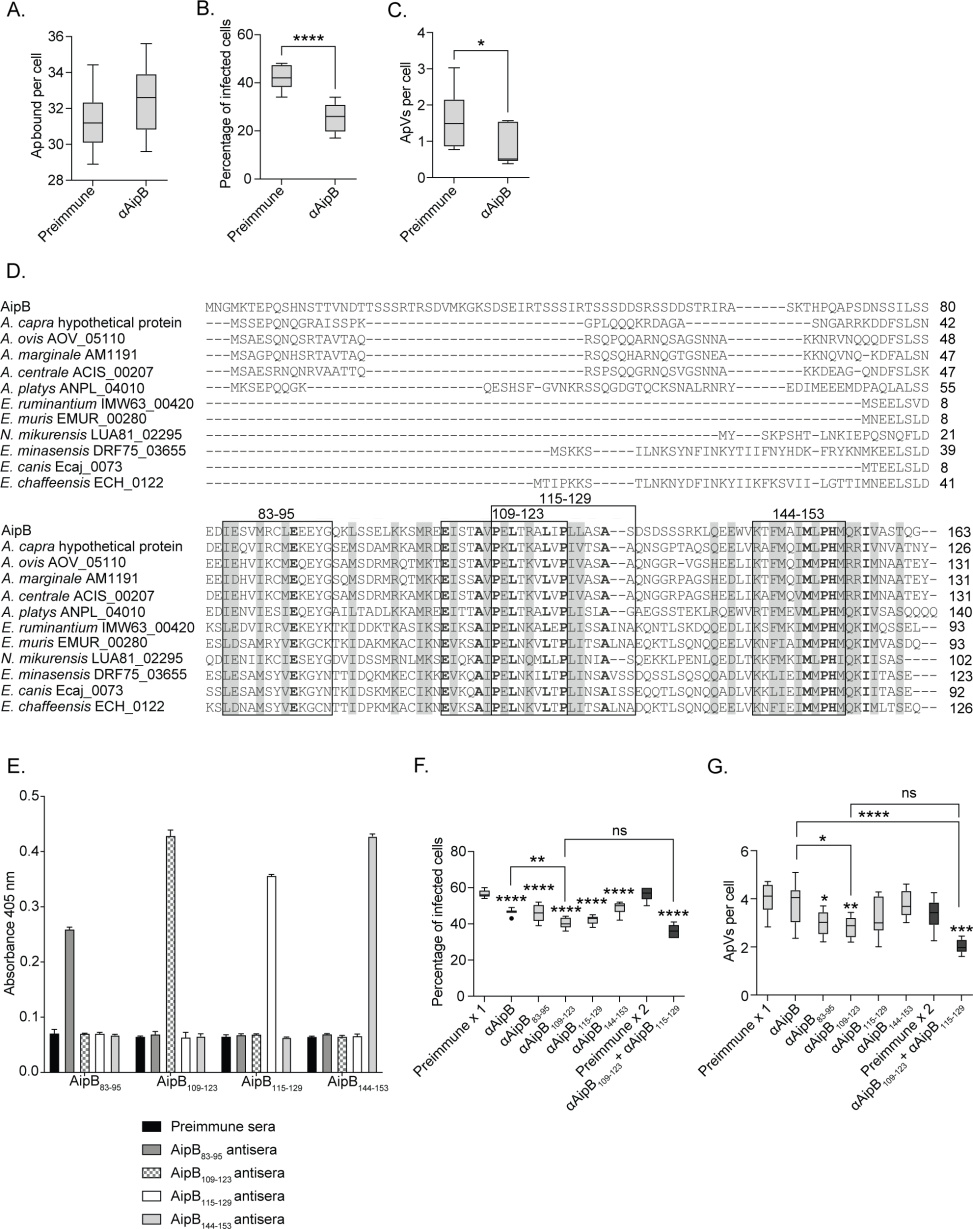
AipB is an *A. phagocytophilum* invasin. (A–C) AipB contributes to *A. phagocytophilum* infection of myeloid cells. *A. phagocytophilum* (Ap) DC organisms were treated with AipB_30-163_ antiserum (αAipB) or preimmune serum prior to incubation with HL-60 cells. The cells were fixed, immunolabeled with *A. phagocytophilum* P44 antibody, and analyzed by immunofluorescence microscopy to determine (**A**) the number of Ap bound per cell at 1 h post-infection. At 24 h post-infection, the percentage of infected cells (**B**) and number of ApVs per cell (**C**) were determined. (**D**) Alignment of AipB with *Anaplasma* and *Ehrlichia* species homologs. Clustal Omega (https://www.ebi.ac.uk/jdispatcher/msa/clustalo) was used to align AipB with *A. capra* hypothetical protein, *A. ovis* Haibei strain AOV_05110, *A. marginale* subspecies centrale Israel strain ACIS_00207, *A. marginale* St. Maries strain AM1191, *A. platys* ANPL_04010, *Ehrlichia ruminantium* IMW63_00420, *E. muris* EMUR_00280, *Neoehrlichia mikurensis* LUA81_02295, *E. minasensi* DRF75_03655, *Ehrlichia canis* Jake strain Ecaj_0073, and *E. chaffeensis* Arkansas strain ECH_0122. Residues conserved in all sequences are in bold text. Gray highlighting demarcates highly similar amino acids. AipB_83-95_, AipB_109-123_, AipB_115-129_, and AipB_144-153_ peptide sequences and corresponding homologous sequences are boxed. (E–G) AipB_109-129_ mediates *A. phagocytophilum* invasion of myeloid cells. (**E**) ELISA in which plates coated with peptides corresponding to AipB_83-95_, AipB_109-123_, AipB_115-129_, and AipB_144-153_ were incubated with the antisera raised against each to verify specificity. Absorbance was read at 405 nm 25 min after the addition of chromogenic substrate. (**F and G**) Ap DC organisms were incubated with rat antisera against AipB_30-163_ (AipB), AipB_83-95_, AipB_109-123_, AipB_115-129_, and AipB_144-153_ or rat preimmune serum, followed by incubation with HL-60 cells. At 24 h, the cells were fixed and immunolabeled with P44 antibody and examined by immunofluorescence microscopy to determine the percentage of infected cells (**F**) and number of ApVs per cell (**G**). Results are indicative of three independent experiments, each performed in triplicate. Data are presented as box-and-whisker plots. Values beyond the upper and lower bounds are outliers indicated with black dots. The horizontal line denotes the median value (50th percentile). The gray boxes contain the 25th to 75th percentiles of the data set. The whiskers extend from the minimum to maximum values. Student’s *t*-test was used to test for a significant difference between pairs. One-way ANOVA with Tukey’s *post hoc* test was used to test for a significant difference among groups. Statistically significant values are indicated (ns, not significant; *, *P* < 0.05; **, *P* < 0.01; ***, *P* < 0.001; ****, *P* < 0.0001).

To determine if AipB contributes to *A. phagocytophilum* infection, isolated DC organisms were treated with antiserum raised against AipB residues 30–163 or preimmune serum and incubated with HL-60 cells. Anti-AipB had no effect on bacterial adherence but reduced the percentage of infected cells and the number of ApVs per cell by 40% and 47%, respectively (Fig. 1A through C). To identify the AipB host cell-interacting domain, we generated antisera against individual 11- to 15-amino acid peptide sequences that are highly conserved among *Anaplasma* and *Ehrlichia* spp. AipB homologs, per the rationale that at least one of these segments would be functionally conserved (Fig. 1D). The targeted peptides were AipB_83-95_, AipB_109-123_, AipB_115-129_, and AipB_144-153_. An enzyme-linked immunosorbent assay (ELISA) was performed to verify the specificity of each antiserum (Fig. 1E). AipB_109-123_ antiserum was the most effective at inhibiting infection, reducing the percentage of infected cells by 28% and the ApVs per cell by 29% (Fig. 1F and G). Antisera against overlapping fragments AipB_109-123_ and AipB_115-129_ inhibited infection comparably, and together did not significantly improve blocking relative to either antiserum alone. Thus, AipB is an *A. phagocytophilum* invasin, and its binding domain lies between residues 109 and 129. All experiments going forward use anti-AipB_109-123_.

### Targeting AipB_109-123_ together with AipA_9-21_, Asp14_113-124_, and OmpA_59-74_ nearly eliminates *A. phagocytophilum* cellular invasion

**Fig 2:**
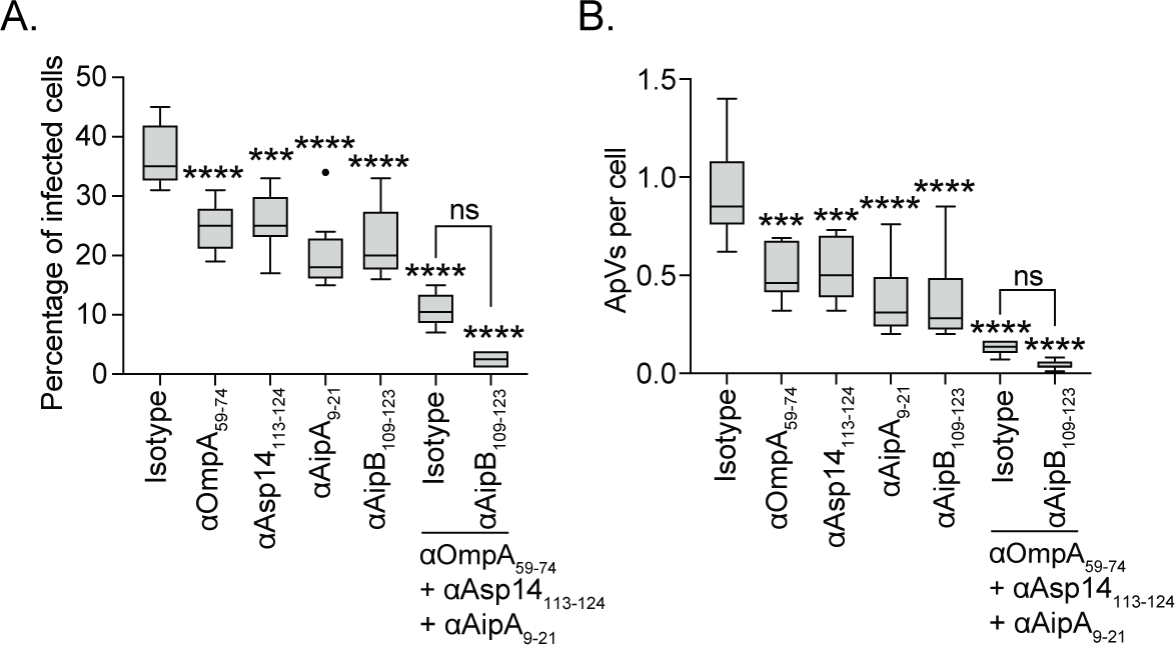
AipB_109-123_ antibody enhances the blocking efficacy of an invasin binding domain antibody cocktail. *A. phagocytophilum* DC bacteria were incubated with rabbit antibodies (α) against OmpA_59-74_, Asp14_113-124_, AipA_9-21_, and AipB_109-123_ or isotype control alone or in the indicated combinations. Treated DCs were incubated with HL-60 cells for 24 h, at which point cells were fixed, immunolabeled with P44 antibody, examined by immunofluorescence microscopy to determine percentage of infected cells (**A**) and number of ApVs per cell (**B**). Data are indicative of three independent experiments performed in triplicate. One-way ANOVA with Tukey’s *post hoc* test was used to test for statistically significant differences among groups. Each condition is compared to the isotype control. Statistically significant values are indicated (ns, not significant; ***P < 0.001, ****P < 0.0001).

Next, the contribution of AipB relative to, and together with, the other known *A. phagocytophilum* surface proteins that mediate infection was assessed. Antisera against AipB_109-123_, OmpA_59-74_, Asp14_113-124_, and AipA_9-21_ each individually reduced infection by 30 to 45% (Fig. 2). Similar to our previous report (19), an antisera cocktail targeting the OmpA, Asp14, and AipA binding domains reduced the percentage of infected cells by 71% and the number of ApVs per cell by 86%. The addition of anti-AipB_109-123_ to this cocktail further reduced infection by 22% and the ApV number per cell by 9%, albeit insignificantly, to nearly abolish infection. Therefore, AipB cooperatively functions with OmpA, Asp14, and AipA, and disrupting the interactions of all four nearly eliminates the ability of *A. phagocytophilum* to invade myeloid cells.

### AipB interacts with the CD18 N-terminus to optimally infect host cells

**Fig 3:**
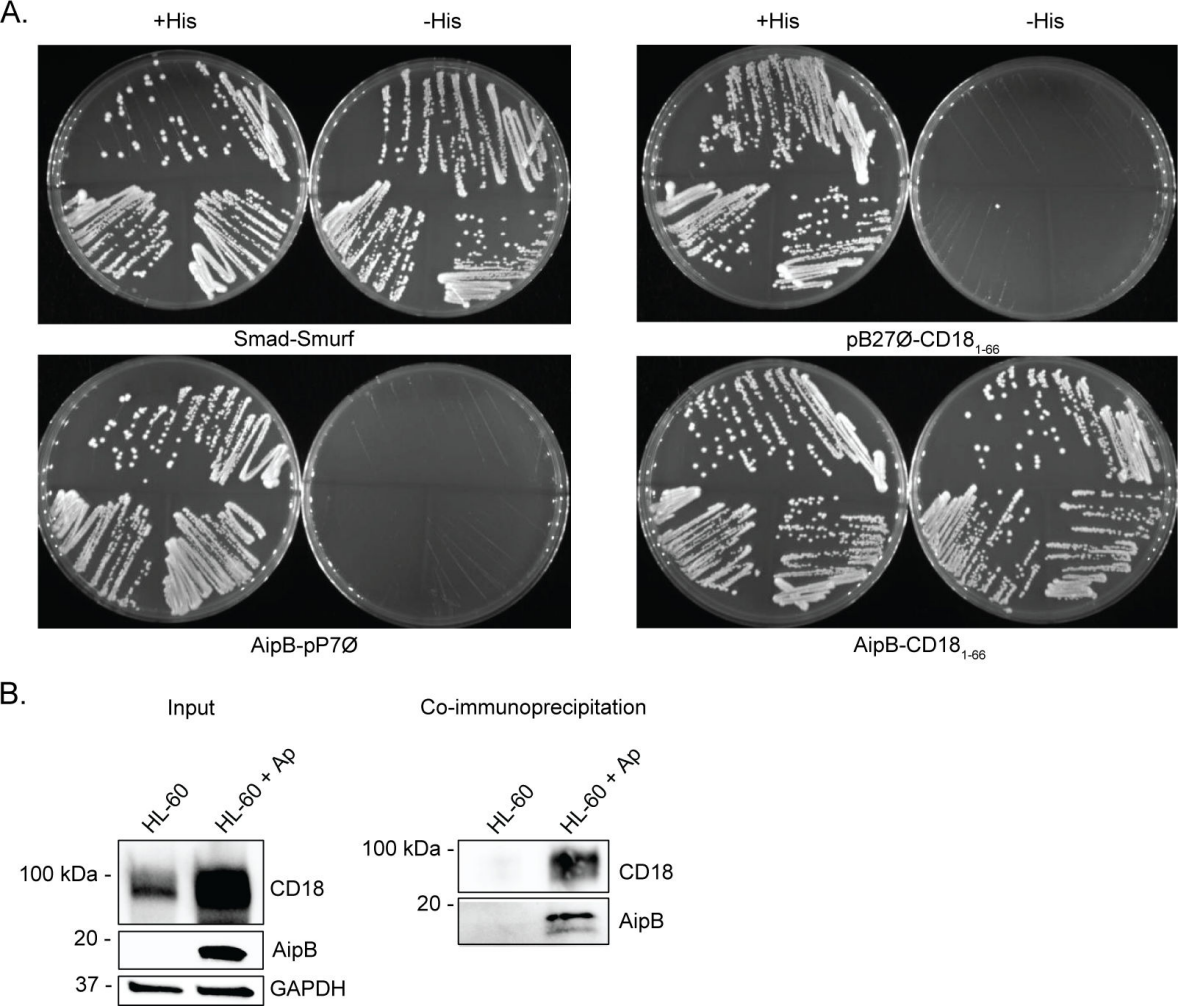
AipB interacts with β2 integrin β-subunit CD18. (**A**) Yeast expressing AipB as bait was mated with yeast expressing CD18_1-66_ as prey. Smad and Smurf were included as a positive control bait-prey pair. Yeast expressing AipB or CD18_1-66_ mated with yeast transformed with empty pP7∅ or pB27∅, respectively, were negative controls. After mating, yeast were streaked onto selective medium with or without histidine. (**B**) HL-60 cells and *A. phagocytophilum* DC organisms were lysed for co-immunoprecipitation. HL-60 lysate alone or HL-60 lysate mixed with DC lysate was incubated with AipB antisera coupled to protein A/G agarose beads to precipitate AipB and its interacting partner CD18. Samples were analyzed by Western blotting using antibodies against CD18, AipB, and glyceraldehyde-3-phosphate dehydrogenase (GAPDH). Results are representative of three independent experiments.

A yeast two-hybrid screen using AipB as bait and a prey human leukocyte cDNA library identified CD18_1-66_ as a potential interacting partner. Of the candidates, CD18 was prioritized for further study because it is expressed exclusively by leukocytes, can be recruited to lipid rafts, can mediate phagocytosis, and is potentially important for *A. phagocytophilum* infection (17, 22, 25, 29, 30, 34, 38–41, 43–45). To further validate the interaction, another yeast two-hybrid assay was performed in which yeast expressing AipB from plasmid pB27∅ as bait or expressing CD18_1-66_ from plasmid pP7∅ as prey were mated. Smad and Smurf were included as a positive control bait-prey pair (46). Yeast expressing AipB or CD18_1-66_ mated with yeast transformed with empty pB27∅ or pP7∅, respectively, were negative controls. Growth on histidine-deficient plates was observed only for transformants carrying both Smad and Smurf or AipB and CD18_1-66_ (Fig. 3A). To validate that endogenous AipB is capable of interacting with CD18, lysates of HL-60 cells or HL-60 cells plus *A. phagocytophilum* DC bacteria were incubated with anti-AipB coupled to agarose beads. CD18 levels were elevated in the mixed lysate input sample, presumably due to upregulation of CD18 that *A. phagocytophilum* infection induces (34) and/or carryover of CD18 bound to DC organisms (Fig. 3B). Immunoprecipitating AipB co-precipitated CD18.

**Fig 4:**
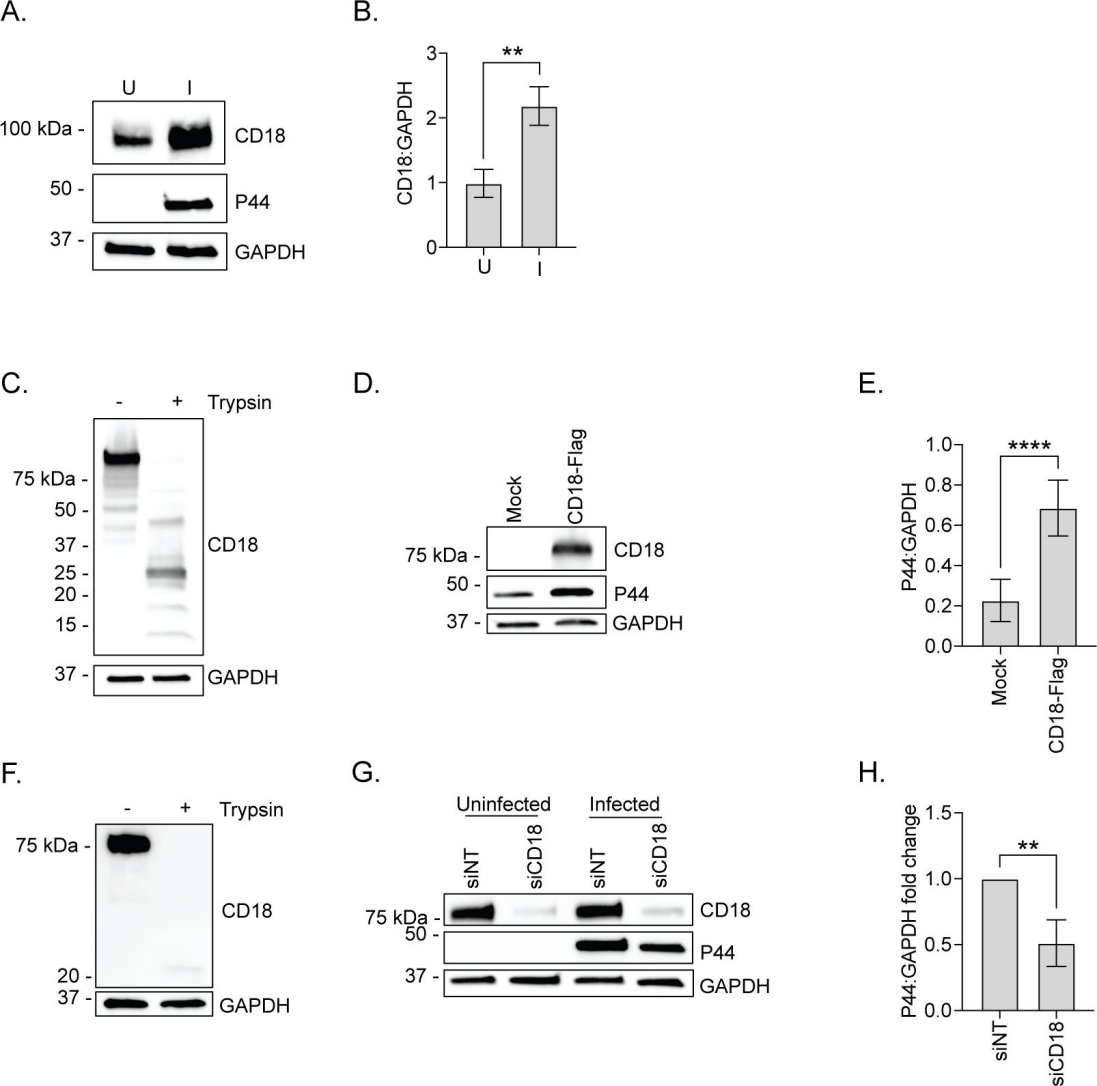
CD18 is important for *A. phagocytophilum* infection. (**A and B**) *A. phagocytophilum* infection upregulates CD18 expression. (**A**) Uninfected (U) and *A. phagocytophilum*-infected (I) HL-60 cells were analyzed by Western blotting using antibodies for CD18, P44, and GAPDH. (**B**) The densitometric signal of CD18 was normalized to that of GAPDH. (C to E) Ectopic expression of CD18-Flag increases host cell permissiveness to *A. phagocytophilum* infection. (**C**) HEK-293T cells transfected to express CD18-Flag were incubated in the presence or absence of trypsin to evaluate CD18-Flag surface expression via Western blotting using antibodies against CD18 and GAPDH. (**D**) HEK-293T cells expressing CD18-Flag were incubated with *A. phagocytophilum* DC organisms. At 24 h post-infection, the cells were analyzed by Western blotting for CD18, P44 (bacterial load), and GAPDH. (**E**) P44 densitometric signal was normalized to that of GAPDH. (F to H) siRNA knockdown of CD18 makes cells less susceptible to *A. phagocytophilum* infection. (**F**) Surface expression of CD18 on RF/6A cells was verified by incubation with trypsin or vehicle control, followed by Western blot analysis using CD18 and GAPDH antibodies. (**G**) RF/6A cells were treated with nontargeting (siNT) or CD18-targeting siRNA or nontargeting siRNA. Western blotting was performed to confirm CD18 knockdown and assess the P44 levels. (**H**) Densitometric signals of P44 were normalized to those of GAPDH. Data are representative of three independent experiments and presented as the mean ± SD. Student’s *t*-test was used to test for statistically significant differences among pairs. Statistically significant values indicated (**, *P* < 0.01; ****, *P* < 0.0001).

**Fig 5:**
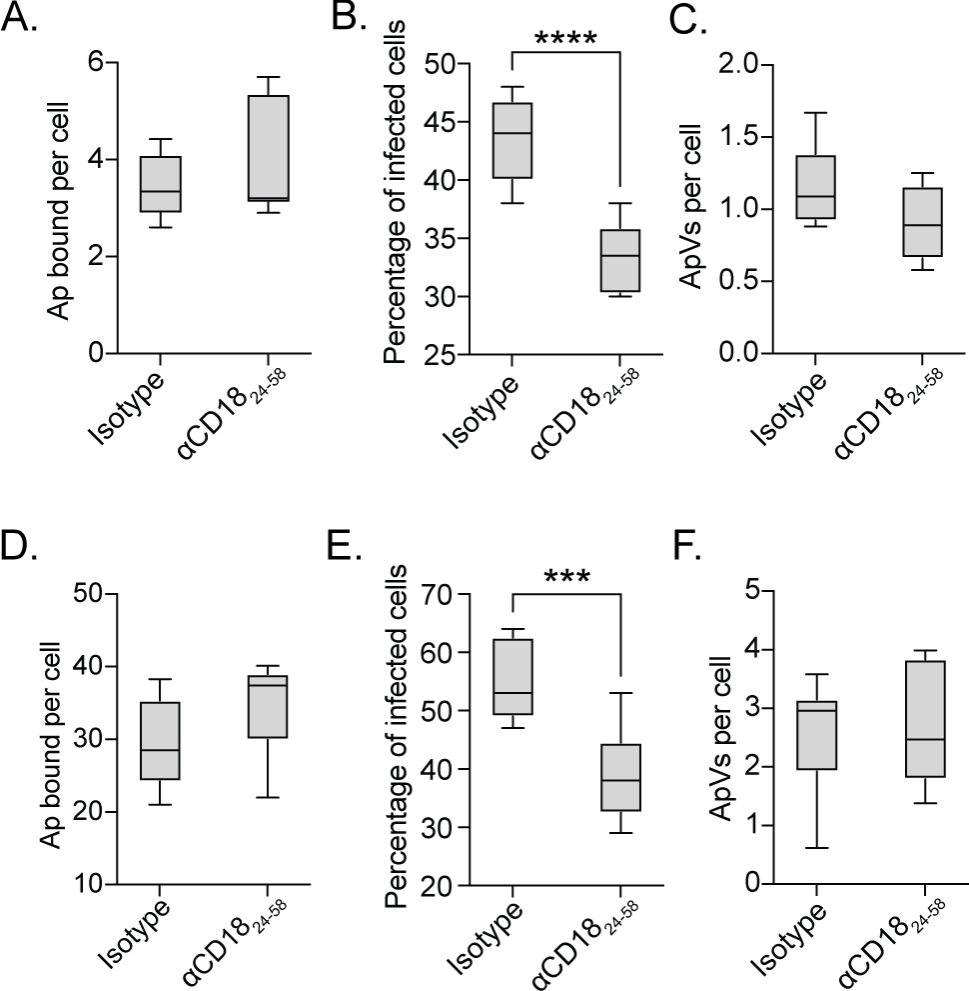
The CD18 N-terminus is important for *A. phagocytophilum* infection. HL-60 cells (A to C) or human neutrophils (D to F) were incubated with αCD18_24-58_ or isotype control, followed by incubation with DC bacteria. Cells were fixed, immunolabeled with P44 antibody, and analyzed by immunofluorescence microscopy at 1 h to determine the number of Ap bound per cell (**A and D**). At 24 h post-infection, the cells were examined to determine the percentage of infected cells (**B and E**) and number of ApVs per cell (**C and F**). Results are indicative of three independent experiments performed in triplicate. Student’s *t*-test was used to test for a statistically significant difference among pairs. Statistically significant values are indicated (***, *P* < 0.001; ****, *P* < 0.0001).

Similar to the upregulation of β2 integrin expression by *A. phagocytophilum*-infected murine neutrophils (34), CD18 levels were elevated twofold in infected HL-60 cells (Fig. 4A and B). To evaluate the relevance of CD18 to *A. phagocytophilum* infectivity, HEK-293T cells, which do not express CD18, were transfected to express full-length CD18 with a C-terminal Flag tag (CD18-Flag) (47). Surface expression of CD18-Flag was verified by susceptibility to trypsin digest (Fig. 4C). Compared to mock-transfected cells, CD18-Flag surface expression conferred more than a threefold increase in the *A. phagocytophilum* load (Fig. 4D and E). To further assess the relevance of CD18 to *A. phagocytophilum* infection, RF/6A cells were used. CD18 surface expression was verified by trypsin digest (Fig. 4F). *A. phagocytophilum* infection of RF/6A cells in which CD18 expression had been knocked down using siRNA was reduced twofold (Fig. 4G and H). Antibody against CD18_24-58_ reduced *A. phagocytophilum* infection of HL-60 cells and human neutrophils by 23% but had no effect on bacterial adherence to either cell type (Fig. 5). Collectively, these data establish that CD18 is an AipB receptor and is important for *A. phagocytophilum* infection.

### Immunization against AipB_109-123_ reduces *A. phagocytophilum* burden in mice

**Fig 6:**
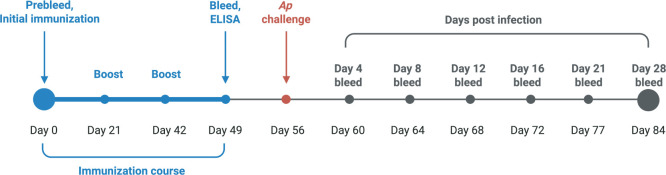
Mouse immunization timeline. On day 0, 13 C57BL/6 mice per group were bled to collect preimmune sera, and initial immunizations were administered via subcutaneous shoulder injection. Mice were boosted on days 21 and 42. On day 46, the mice were bled and sera were collected for ELISA to verify the presence of peptide-specific antibodies. Mice were challenged on day 56 with 1 × 10^8^ *A. phagocytophilum* DC organisms. Mice were bled on days 4, 8, 12, 16, and 21 and sacrificed on day 28. Peripheral blood smears were microscopically examined for neutrophils containing ApVs to assess bacterial load.

**Fig 7:**
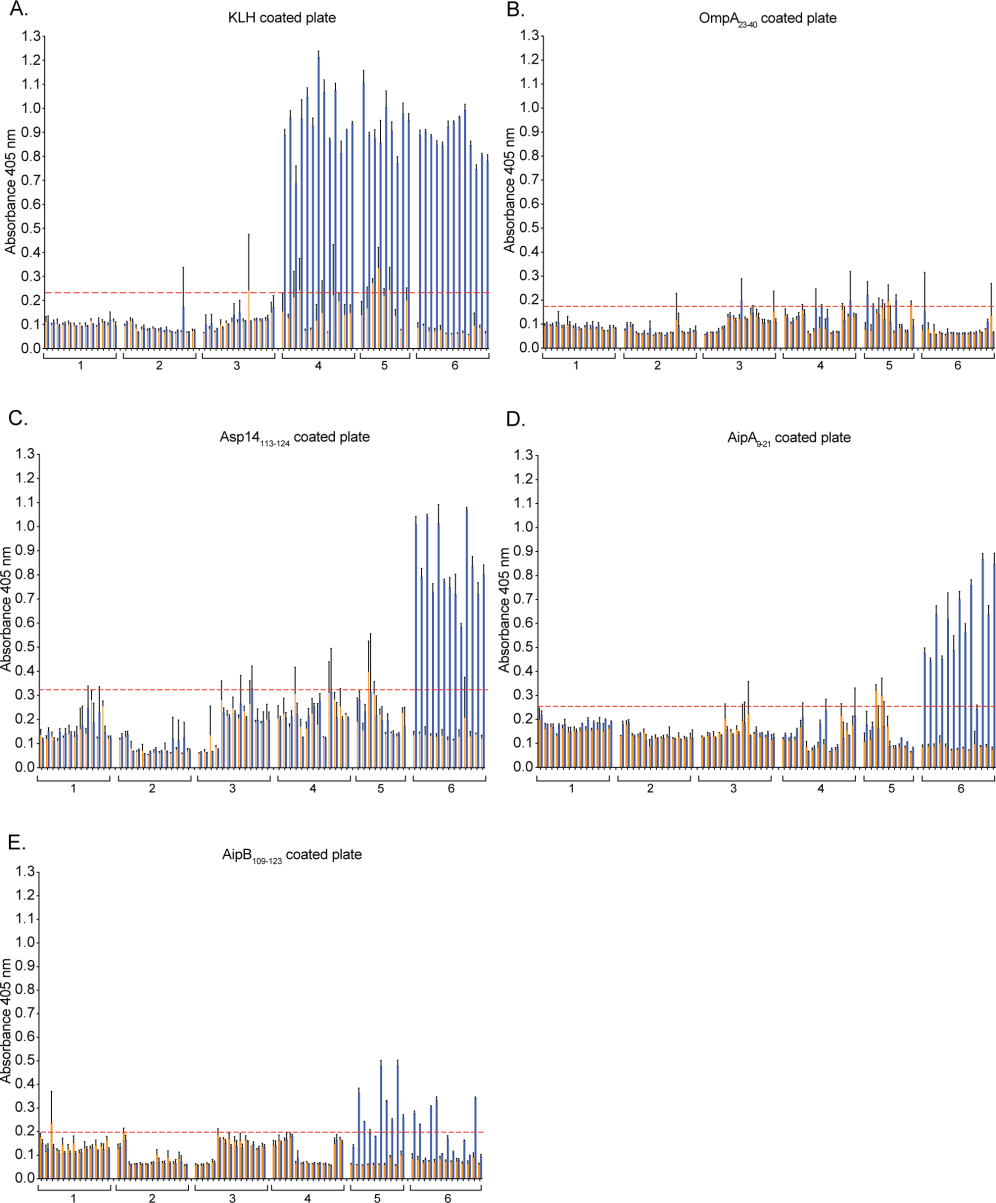
Immunization of mice against AipB_109-123_, Asp14_113-124_, and AipA_9-21_ elicits humoral immune responses. Preimmune sera (orange bars) or sera collected on day 49 (blue bars) from each mouse were added to wells coated with (**A**) KLH, (**B**) OmpA_23-40_, (**C**) Asp14_113-124_, (**D**) AipA_9-21_, or (**E**) AipB_109-123_. Numbers indicate treatment groups: no immunization, no challenge (1), no immunization, challenge (2), alum, challenge (3), alum and KLH, challenge (4), alum and KLH-AipB109-123 (5), alum and KLH-Asp14_113-124_ + KLH-AipA_9-21_ + KLH-AipB_109-123_, challenge (6). Sera from individual mice were analyzed in triplicate by ELISA. Absorbance was read at 405 nm, 25 min after the addition of chromogenic substrate. Dotted lines indicate two times the average absorbance for preimmune sera.

Immunizing mice against keyhole limpet hemocyanin (KLH)-conjugated Asp14_113-124_ and/or AipA_9-21_ elicits partial protection against *A. phagocytophilum* infection, while KLH-OmpA_54-79_ is non-immunogenic (48). To evaluate the relevance of AipB to infection *in vivo*, C57BL/6 mice were immunized against KLH-AipB_109-123_ alone or in combination with KLH-Asp14_113-124_ and KLH-AipA_9-21_ in alum. The six experimental groups, consisting of 13 mice each, were (i) no immunization or bacterial challenge; (ii) no immunization followed by challenge with *A. phagocytophilum*; (iii) injection with alum followed by *A. phagocytophilum* challenge; (iv) injection with KLH and alum followed by bacterial challenge; (v) injection with KLH-AipB_109-123_ in alum followed by *A. phagocytophilum* challenge; and (vi) injection with KLH-AipB_109-123_, KLH-Asp14_113-124_, and KLH-AipA_9-21_ followed by *A. phagocytophilum* challenge (Table 1). Mice were boosted on days 21 and 42 (Fig. 6). To test if the immunization procedure elicited humoral immune responses specific for each antigen, serum samples collected on day 49 were used to screen ELISA plates coated with KLH or unconjugated AipB_109-123_, Asp14_113-124_, or AipA_9-21_. OmpA_23-40_ served as a negative control, as it was not used as an immunogen and has been verified to be irrelevant for *A. phagocytophilum* infection (19, 48). Serum samples from mice immunized with KLH-conjugated peptides recognized KLH and the specific invasin binding domain immunogen per group but not the other two binding domain peptides or OmpA_23-40_ (Fig. 7). Sera from control groups 1–3 that had not been immunized against any of the invasin binding domain peptides failed to recognize any peptide.

**TABLE 1:** Experimental groups used in immunization study

Group	Immunization	Peptide(s)	Infection*^a^*
1	No immunization	N/A^*b*^	No
2	No immunization	N/A	Yes
3	Alum	N/A	Yes
4	Alum and KLH	N/A	Yes
5	Alum and KLH-conjugated peptide	AipB_109-123_	Yes
6	Alum and KLH-conjugated peptide	Asp14_113-124_, AipA_9-21_, AipB_109-123_	Yes

^*a*^ Mice were challenged interperitoneally with 1 × 10^8^* A. phagocytophilum* organisms on day 56.

^*b*^ N/A, not applicable.

**Fig 8:**
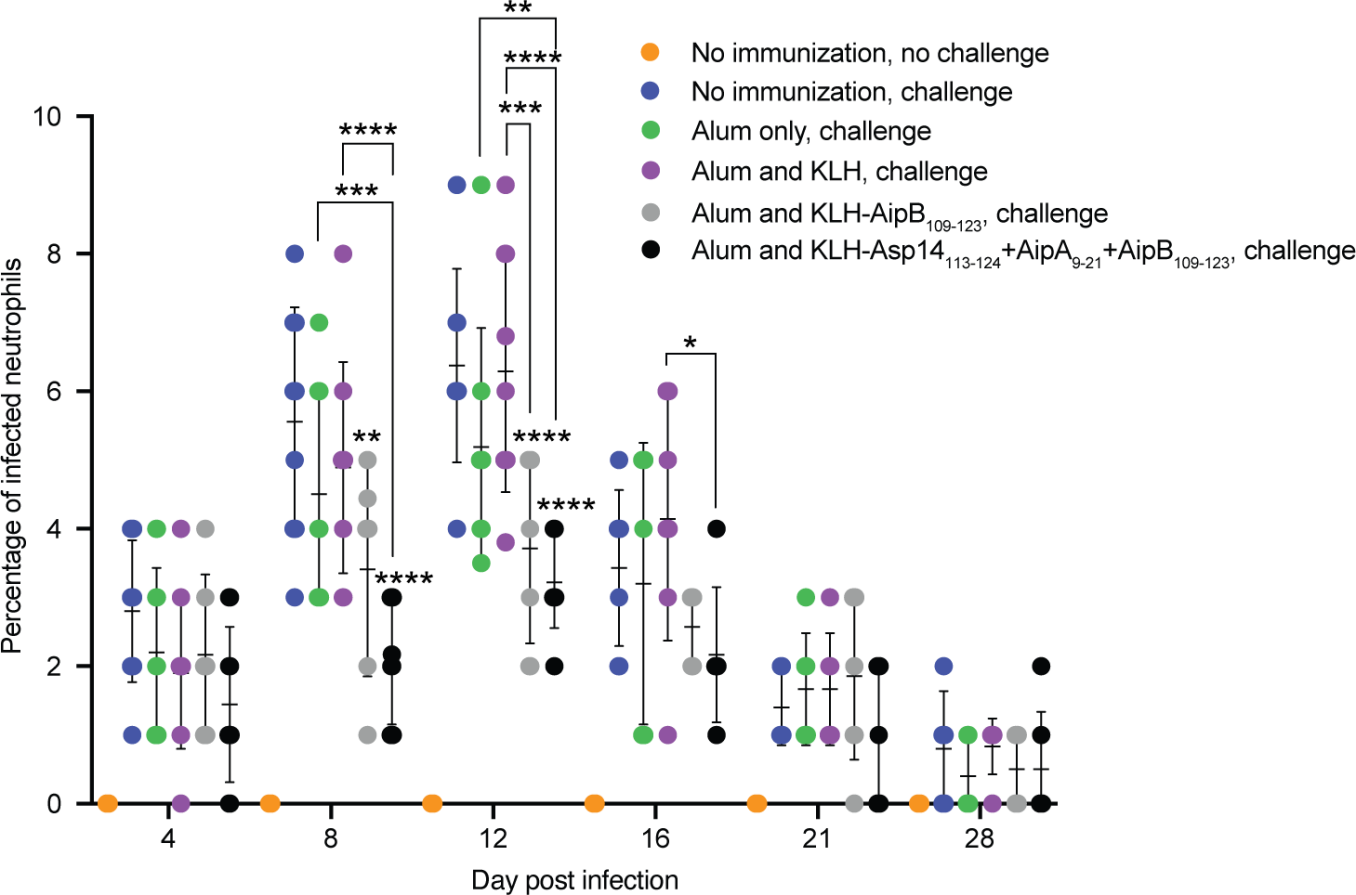
Mice immunized against AipB_109-123_ alone or together with Asp14_113-124_ and AipA_9-21_ are resistant to *A. phagocytophilum* infection. C57BL/6 mice immunized against KLH-AipB_109-123_, KLH-AipB_109-123_ + KLH-Asp14_113-124_ + KLH-AipA_9-21_, or the indicated controls were inoculated with *A. phagocytophilum* DC organisms. Peripheral blood samples drawn on the indicated days were examined by light microscopy for *A. phagocytophilum*-infected neutrophils. Each dot corresponds to the percentage of infected neutrophils per mouse, as determined by examining at least 100 neutrophils. Two-way ANOVA with Tukey’s *post hoc* test was used to test for significant differences among groups on each day post-infection. Data are presented as the mean ± SD determined for ten mice per group. Significance was determined relative to the no immunization, challenge group unless denoted by brackets. Statistically significant values are indicated (*, P < 0.05; **, *P* < 0.01; ***, *P* < 0.001; ****, *P* < 0.0001).

On day 56, the mice were inoculated with *A. phagocytophilum* DC organisms. The bacterial peripheral blood burden was evaluated on days 4, 8, 12, 16, 21, and 28 post-infection by examining blood smears for the percentage of infected neutrophils. The *A. phagocytophilum* burden peaked on day 12 and began to wane thereafter in all challenged mice (Fig. 8). The bacterial load was comparable for non-immunized mice and mice that had been mock-immunized with either alum or alum and KLH. Relative to non-immunized mice, the bacterial burden was comparably reduced approximately twofold in mice immunized against KLH-AipB_109-123_ alone and together with KLH-Asp14_113-124_ and KLH-AipA_9-21_. Thus, AipB_109-123_, like Asp14_113-124_ and AipA_9-21_, is important for *A. phagocytophilum* to productively infect mice, and immunizing against it alone provides a partially protective immune response similar to that achieved by immunizing against all three invasin binding domains.

## DISCUSSION

*A. phagocytophilum* is the first rickettsial pathogen known to interact with a β2 integrin to promote cellular invasion. Our findings establish that *A. phagocytophilum* AipB binding to the β2 integrin β-subunit, CD18, mediates bacterial uptake and demonstrates the invasin’s importance to productive infection *in vivo*. Specifically, a linear binding domain within AipB_109-129_ interacts with CD18 within residues 1–66. Because the human CD18 signal peptide that gets cleaved consists of amino acids 1–22, the relevant region can be narrowed down to residues 23–66. This is supported by the ability of anti-CD18_24-58_ to disrupt *A. phagocytophilum* infection of host cells. Other intracellular bacterial, parasitic, and fungal pathogens also use β2 integrins as receptors for infection (49–60). Such conservation is likely linked to the phagocytic uptake that β2 integrin engagement induces. For instance, the binding of AipB and *Mycobacterium tuberculosis* mammalian cell entry protein Mce3C to CD18 are both critical for cellular invasion but not adherence (50). *A. phagocytophilum* binding elicits actin cytoskeletal changes and signaling through Src kinase, Syk, and ROCK1, which also occurs when Mce3C binds β2 integrins (17, 41, 61). Hence, the AipB-CD18 interaction could analogously induce signaling that helps facilitate *A. phagocytophilum* uptake. Interestingly, the major ligand-binding domain for β2 integrins lies within the I-like domain of the α-subunit (62). Yet, AipB and Mce3C target the β-subunit (50), suggesting that CD18 might be capable of initiating outside-in signaling independent of the α-subunit. Indeed, HEK-293T cells transfected to express CD18 alone exhibit significantly increased susceptibility to *A. phagocytophilum* infection despite the lack of heterodimer formation. Alternatively, ectopically expressed CD18 on HEK-293T cells could allow for increased *A. phagocytophilum* binding that is followed by uptake via a separate mechanism, such as that which is dependent on PDI. Notably, PDI is ubiquitously expressed and therefore the only other known *Anaplasma* receptor naturally expressed on these cells.

Engagement of PSGL-1 on neutrophils increases β2 integrin expression and enhances β2 integrin-mediated adhesion (63). After docking to the human neutrophil surface via binding to PSGL-1 (64), *A. phagocytophilum* rapidly promotes β2 integrin expression and PSGL-1 shedding (40). In the absence of PSGL-1, β2 integrin alone is insufficient to mediate neutrophil arrest on endothelium (63), a phenotype that neutrophils exhibit upon *A. phagocytophilum* binding (39, 40). It has been proposed that PSGL-1 shedding maintains *A. phagocytophilum*-infected neutrophils in circulation to maximize their acquisition by ticks and thus continuation of the enzootic cycle (40). Perhaps by simultaneously upregulating CD18, the bacterium ensures that it has a key receptor for invasion once PSGL-1 has been shed. Binding to β2 integrin can stimulate nicotinamide adenine dinucleotide phosphate (NADPH) oxidase assembly for microbicidal ROS production (35). *A. phagocytophilum* binding to neutrophils promotes NADPH oxidase assembly (65), and infected neutrophils from CD11b^-/-^ mice exhibit a modest increase in bacterial burden compared to wild-type mice (34). Both observations support that CD11b/CD18 may partially control the infection, at least initially (34). Ligation of CD13, another *A. phagocytophilum* receptor, also stimulates ROS production (17, 66). Yet, *A. phagocytophilum* overcomes this response as it scavenges extracellularly released superoxide during binding and entry, replicates in a pathogen-modified multivesicular body that excludes NADPH oxidase, and impairs expression of NADPH oxidase components to render its host neutrophil incapable of ROS-dependent killing (12, 65, 67–69).

Based on the results herein combined with our and others’ previous reports (11, 15, 18–20, 64, 70–72), it is likely that *A. phagocytophilum* initially docks itself to the human neutrophil surface by binding sLe^x^-capped PSGL-1 N-terminus using OmpA and possibly an additional adhesin, followed by the Asp14-PDI, AipA-CD13, and AipB-CD18 interactions that promote entry. Anti-AipB_109-123_ partially inhibits *A. phagocytophilum* infection of myeloid cells to a degree comparable to that achieved by individual antisera against OmpA_59-74_, Asp14_113-124_, or AipA_9-21_. Adding anti-AipB_109-123_ to an OmpA_59-74_-Asp14_113-124_-AipA_9-21_ antibody cocktail boosts the blocking efficacy from 71% to 93%, indicating that AipB cooperates in a multi-adhesin/invasin network to promote *Anaplasma* invasion. AipB_109-123_ antibodies also significantly impair *A. phagocytophilum* infection of neutrophils *in vivo*. Yet, immunizing against Aip_109-123_ alone is just as protective as immunizing against it together with AipA_9-21_ and Asp14_113-124_. Similarly, immunizing against Asp14_113-124_ or AipA_9-21_ alone yielded responses that were just as effective as immunizing against both binding domains (48). This could be due to Asp14-, AipA-, and/or AipB-receptor interactions converging to activate the same signaling pathway to promote *A. phagocytophilum* uptake. Src is robustly activated by *A. phagocytophilum* binding and is essential for infection (17). Ligation of CD13 and CD18 each activates Src, and PDI is indirectly linked to Src activation per its role in regulating β2 integrin function (21, 22, 24). The lack of complete protection in mice immunized against Asp14, AipA, and AipB could be due to *A. phagocytophilum* utilizing an additional unidentified host receptor to invade murine neutrophils or to the bacterium differentially utilizing known receptors to infect murine versus human neutrophils. For instance, *A. phagocytophilum* cannot bind murine PSGL-1, and it is therefore irrelevant for infection in mice (70, 71). Alternatively, the level of circulating antibody may have been inadequate, or additional non-humoral immune responses for maximally controlling *A. phagocytophilum* infection *in vivo* might not have been sufficiently induced.

Overall, we established that the AipB-CD18 interaction is part of a multi-*A. phagocytophilum*-receptor complex that cooperatively facilitates bacterial invasion of human and murine neutrophils alike. The interaction is critical for optimal infection *in vivo*. AipB_109-123_, together with AipA_9-21_ and Asp14_113-124_, is an immunogen that elicits partial protection in mice. Because antibodies against the three binding domains impede *A. phagocytophilum* entry into myeloid cells, which is an essential step for this obligate intracellular bacterium’s survival, they could potentially be included in a multivalent vaccine. To achieve maximal protection, however, additional *A. phagocytophilum*-receptor interactions may need to be identified and/or immunization approaches should ideally elicit high titers of circulating antibodies as well as other immune responses proven to be critical for eliminating infection, including CD4^+^ T cell responses, dendritic cell maturation, and IFN-γ production (17, 73–75). This study represents a foundational step toward this goal.

## MATERIALS AND METHODS

### Cell lines and cultivation of *A. phagocytophilum*

The *A. phagocytophilum* NCH-1 strain, which was originally isolated from a patient in Nantucket, MA (76), was used for all infection studies. Human embryonic kidney HEK-293T cells (CRL-3216; American Type Culture Collection [ATCC]) and uninfected and *A. phagocytophilum*-infected RF/6A *Macaca mulatta* chorioretinal cells (CRL-1780; ATCC) were cultured in Dulbecco’s Modified Eagle’s Medium (Thermo Fisher Scientific) supplemented with 10% heat-inactivated fetal bovine serum (FBS; Gemini Bio-Products), 15 mM HEPES (Affymetrix), and 1× MEM Non-Essential Amino Acids (Invitrogen). Uninfected and *A. phagocytophilum*-infected human promyelocytic HL-60 cells (CCL-240; ATCC) were cultured in Iscove’s Modified Dulbecco’s Medium (Invitrogen) supplemented with 10% FBS (IMDM-10). All cells were grown in a humidified incubator at 37°C with 5.0% CO_2_ (12, 77, 78).

### Antisera generation

Rabbit mono-specific antisera against OmpA_59-74_, Asp14_113-124_, and AipA_9-21_ and mouse antiserum against AipB residues 30–163 were generated previously (20, 48). Rabbit antiserum against full-length AipB was kindly provided by Dr. Erol Fikrig of Yale University (28). Peptides corresponding to AipB_83-95_, AipB_109-123_, AipB_115-129_, and AipB_144-153_ were synthesized and conjugated to KLH at a 1:1 molar ratio by Biosynth. Fifty micrograms each of these four KLH-conjugated peptides was emulsified in a 1:1 ratio with complete Freund’s adjuvant and administered in a total volume of 400 μL to 8-week-old Sprague-Dawley rats. At weeks 3 and 5, the rats were boosted with 25 μg of KLH-conjugated peptide in incomplete Freund’s adjuvant. At week 6, the rats were euthanized by CO_2_ asphyxiation, blood was collected by cardiac puncture, and the antisera recovered. Rabbit mono-specific antiserum against KLH-AipB_109-123_ was generated by Biosynth. All peptide antisera specificity was confirmed by ELISA as described below.

### Neutrophil infections

Neutrophils were isolated, and purity was confirmed as previously described (17, 79). Neutrophils were incubated with isolated *A. phagocytophilum* DC organisms isolated from four times as many infected HL-60 cells.

### Antibody inhibition of *A. phagocytophilum* infection

To evaluate the relevance of AipB and CD18 to *A. phagocytophilum* binding and infection, inhibition assays utilizing antibodies were performed. For inhibition assays with CD18 antibodies, HL-60 cells or neutrophils were incubated with 10 μg mL^−1^ of polyclonal anti-integrin β2 (CD18_24-58_) (Boster Biological [PA1124]) or rabbit IgG (Invitrogen [02-6102]) for 1 h in a humidified incubator at 37°C with 5% CO_2_ and inverted every 10 min. Host cells were subsequently incubated with isolated *A. phagocytophilum* DC organisms from two to four times as many infected HL-60 cells as previously described (17). DCs were incubated with heat-inactivated antisera against full-length AipB or preimmune sera at a 1:5 dilution for 1 h. To narrow down the AipB binding domain, DC bacteria were incubated with 100 μg mL^−1^ of heat-inactivated AipB_83-95_, AipB_109-123_, AipB_115-129_, or AipB_144-153_ antisera or preimmune sera equal in volume to the largest amount of AipB antisera used. To evaluate the efficacy of an invasin binding domain antibody cocktail, *A. phagocytophilum* DCs were treated with 100 μg mL^−1^ of OmpA_59-74_, Asp14_113-124_, AipA_9-21_, and AipB_109-123_ antibodies alone or in combinations. Rabbit IgG (Invitrogen [02-6102]) served as a negative control. In each blocking experiment, DC organisms were incubated with HL-60 cells for 1 h in a humidified incubator at 37°C with 5% CO_2_ and inverted every 10 min. Cells were washed two to three times with phosphate-buffered saline (PBS) to remove unbound bacteria and plated in IMDM-10 in a humidified incubator at 37°C with 5% CO_2_. Bacterial binding was evaluated at 1 h post-infection and the percentage of infected cells at 24 h post-infection by indirect immunofluorescence microscopy as described below.

### Indirect immunofluorescence microscopy

*A. phagocytophilum*-infected host cells were analyzed by indirect immunofluorescence assay as previously described (17). Briefly, *A. phagocytophilum*-infected HL-60 cells were cytocentrifuged onto glass slides in a Shandon Cytospin 4 centrifuge (Thermo Electron). Cells were fixed in Hema 3 fixative (Thermo Fisher Scientific) for 5 min, followed by incubation with *A. phagocytophilum* P44 antisera (12) (1:500) and Alexa Fluor 594-conjugated chicken anti-rabbit IgG (1:1,000; Invitrogen [A21442]). Coverslips were mounted with ProLong Gold Antifade mounting media plus DAPI (Invitrogen). To evaluate bacterial binding, 50 cells per slide were examined at 1 h post-infection. The percentage of infected cells and the number of ApVs per cell were determined at 24 h post-infection by evaluating 100 cells per slide. Slides were examined using an Olympus BX51 spinning disk confocal microscope (Olympus).

### Yeast two-hybrid analysis

ULTimate yeast two-hybrid analysis was performed by Hybrigenics Services (https://www.hybrigenics-services.com/). The mammalian codon-optimized DNA sequence encoding *A. phagocytophilum* AipB (bait; APH_1235 in HZ strain genome [https://www.ncbi.nlm.nih.gov/protein/88597948]) was introduced into yeast and mated with yeast containing a randomly primed human activated leukocyte cDNA library (prey). Prey fragments from positively selected clones were PCR-amplified, sequenced, and identified using the NCBI GenBank Database (https://www.ncbi.nlm.nih.gov/nucleotide/) and Basic Local Alignment Search Tool (https://blast.ncbi.nlm.nih.gov/Blast.cgi). The predicted biological score was calculated to assess the reliability of each interaction, ranging from the highest (A score) to the lowest (E score) probability of specificity between two proteins (80). To further evaluate the AipB-CD18 interaction, Hybrigenics performed the following screen. Yeast expressing AipB from plasmid pB27∅ as bait or expressing CD18_1-66_ from plasmid pP7∅ as prey were mated. Smad and Smurf were included as a positive control bait-prey pair (46). Yeast expressing AipB or CD18_1-66_ were mated with yeast transformed with empty pB27∅ or pP7∅, respectively, as negative controls. The transformants were streaked onto selective medium with and without histidine. Growth on histidine-deficient plates indicated that protein-protein interaction had occurred.

### Co-immunoprecipitation assay

To verify the AipB-CD18 interaction, a co-immunoprecipitation assay was performed using HL-60 cells and naturally released *A. phagocytophilum* DC organisms purified from the supernatant of HL-60 cells using a 2 µm filter. Cells were lysed on ice using immunoprecipitation lysis buffer (50 mM Tris base, 150 mM NaCl, 1 mM EDTA, 0.5% NP-40, and 10% glycerol) for 1 h. Protein concentrations were determined by Bradford assay. To preclear lysates, protein A/G agarose (Thermo Fisher Scientific) was washed twice in immunoprecipitation lysis buffer, centrifuged at 8,600 × *g* for 30 s, and added to at least 150 μg of HL-60 cell and/or *A. phagocytophilum* DC lysates in a final volume of 300 µL. Samples were incubated on a rotator at 4°C for 2 h, followed by centrifugation at 8,600 × *g* for 30 s. Ten microliters of murine AipB antisera (20) was added to the supernatant from each sample and rotated at 4°C overnight. Pre-washed protein A/G agarose beads were added to each tube and incubated on a rotator for 2 h at 4°C. Samples were centrifuged at 8,600 × *g* for 30 s to pellet the beads and washed four times with immunoprecipitation buffer. Washed beads were resuspended in Laemmli buffer (Bio-Rad) with 500 mM imidazole (Thermo Fisher Scientific) and incubated at 100°C for 10 min to elute bound proteins. Input lysates (10 μg) and eluates were resolved by SDS-PAGE and screened by Western blotting.

### Mammalian cell transfections

To evaluate the relevance of CD18 to *A. phagocytophilum* infection, HEK-293T cells grown to confluency in six-well plates were transfected with 4 μg of plasmid CD18-Flag (Sino Biological) using Lipofectamine 2000 (Invitrogen) following the manufacturer’s directions. At 18 h, transfected cells were incubated with *A. phagocytophilum* DC bacteria naturally released from RF/6A cells into the culture media as previously described (13). Cells were harvested by scraping, centrifuged at 5,200 × *g* for 15 min, lysed using radioimmunoprecipitation assay buffer (50 mM Tris-HCl [pH 7.4], 150 mM NaCl, 1% NP-40, 1% sodium deoxycholate, 1 mM EDTA [pH 8]) supplemented with Halt Protease and Phosphatase Inhibitor Cocktail (Thermo Fisher Scientific; 1:100), and analyzed by Western blot for CD18-Flag, P44, and GAPDH as described below. Surface expression of CD18-Flag on HEK-293T cells was verified by incubating transfected cells with 200 μL of 0.05% (vol/vol) trypsin (Gibco) in PBS or PBS for 10 min at 37°C, followed by the addition of 800 μL of media to each sample to deactivate trypsin. Cells were washed twice with PBS and lysed for Western blot analysis.

CD18 surface expression on RF/6A cells was evaluated by trypsin digest as described above. To assess the contribution of CD18 to *A. phagocytophilum* infection of RF/6A cells, the cells were grown to 80% confluency in six-well plates. Five µM ON-TARGETplus human *ITGB2* (CD18 mRNA) siRNA (Dharmacon) or non-targeting control siRNA (Dharmacon) were prepared with Lipofectamine RNAiMax Reagent (Invitrogen) per the manufacturer’s protocol. At 72 h, the cells were infected with *A. phagocytophilum* DCs that had been naturally released from RF/6A cells by centrifuging the plates at 1,000 × *g* for 3 min. After a 1 h incubation at 37°C, unbound bacteria were washed off. At 24 h post-infection, cells were collected by scraping, lysed, and assessed for P44 and GAPDH expression by Western blot analysis as described below.

### Western blotting

Protein lysates of uninfected and infected HL-60 cells, RF/6A cells, and HEK-293T cells were analyzed by Western blotting as described previously (17). Primary antibody dilutions, following the manufacturer’s recommendations or based on prior studies, targeted *A. phagocytophilum* P44 (12) (1:5,000), CD18 (D4N5Z; 1:1,000; Cell Signaling Technology [73,663]), and GAPDH (G-9; 1:750; Santa Cruz [sc-365062]). Secondary antibodies were horseradish peroxidase (HRP)-linked anti-mouse IgG (1:10,000; Cell Signaling Technology [7076S]) or anti-rabbit IgG (1:10,000; Cell Signaling Technology [7074S]). Blots were incubated with SuperSignal West Pico PLUS chemiluminescent substrate (Thermo Fisher Scientific) following the manufacturer’s directions and imaged using the ChemiDoc Touch Imaging System (Bio-Rad). The densitometric signals of P44 and CD18 were normalized to those of GAPDH from the same sample using Image Lab 6.1 (Bio-Rad).

### Mouse studies

Experiments were performed with 5-week-old male C57BL/6 mice (Jackson Laboratories) since they experience greater susceptibility to *A. phagocytophilum* infection than female mice (81). Groups of 13 mice were injected with immunogen or control. Tail bleeds were performed on day 0 to collect preimmune sera, and mice received subcutaneous shoulder injections of Imject Alum adjuvant (Thermo Fisher Scientific); adjuvant and KLH, adjuvant and KLH-AipB_109-123_; or adjuvant and a cocktail of equal parts AipB_109-123_, Asp14_113-124_, and AipA_9-21_ conjugated to KLH. Mice receiving KLH were injected with 100 μg of protein. Mice injected with KLH-peptide conjugates received 100 μg of KLH-AipB_109-123_ or 100 μg of KLH-AipB_109-123_/Asp14_113-124_/AipA_9-21_. Mice were boosted on days 21 and 42. On day 49, tail bleeds were performed to collect sera for ELISA. On day 56, all mice except no-challenge controls were inoculated with 1 × 10^8^ *A. phagocytophilum* DC organisms via intraperitoneal injection (48). Tail bleeds were performed on days 4, 8, 12, 16, and 21 post-infection, and mice were euthanized and blood collected by cardiac puncture on day 28. Citrate-dextrose solution (Sigma-Aldrich) at 100 U mL^−1^ was added to blood to prevent coagulation. Blood smears were examined microscopically for the presence of infected neutrophils as described previously (48).

### Indirect ELISA

To evaluate humoral response in immunized mice, 96-well plates were coated with 500 ng per well of KLH or unconjugated peptides corresponding to AipB_109-123_, Asp14_113-124_, AipA_9-21_, and OmpA_23-40_ in carbonate buffer (15 mM sodium carbonate, 34.88 mM sodium bicarbonate, pH 9.6) overnight at 4°C. Plates were blocked for 3 h at room temperature with 5% (wt/vol) non-fat dry milk in PBS with 0.5% (vol/vol) Tween 20 (PBS-T). The plates were then incubated with sera from immunized mice at 1:100 in 5% non-fat dry milk in PBS-T for 1 h at room temperature, washed three times with PBS-T, incubated with HRP-linked anti-mouse IgG (1:15,000; Cell Signaling Technology [7076S]), and then washed again. Chromogenic substrate (400 μL of 194.3 mM ABTS [2,2’-azino-bis(3-ethylbenzothiazoline-6-sulfonic acid) diammonium salt] substrate [Sigma-Aldrich], 9.6 mL citrate buffer [10.4 mM, pH 4.1], 10 μL hydrogen peroxide [Sigma-Aldrich]) was added to each well for 25 min, followed by absorbance reading at 405 nm in an ELx808 plate reader (Fisher Scientific) (48). To verify the specificity of AipB peptide antisera, 96-well plates were coated with 500 ng per well of unconjugated AipB_83-95_, AipB_109-123_, AipB_115-129_, or AipB_144-153_ peptides. Plates were incubated with sera against KLH-conjugated AipB_83-95_, AipB_109-123_, AipB_115-129_, and AipB_144-153_. The plates were washed, incubated with HRP-linked anti-rat IgG (1:15,000; Cell Signaling Technology [7077S]), washed again, incubated with chromogenic substrate for 25 min, and absorbance at 405 nm was read.

### Statistical analysis

GraphPad Prism version 9 (San Diego, CA) was used for all statistical analyses. Student’s *t*-test was used to test for significant differences between paired data. One-way analysis of variance (ANOVA) or two-way ANOVA with Tukey’s *post hoc* test was used to evaluate significant differences among groups. *P*-values <0.05 were considered statistically significant.
